# *Mycobacterium bovis* bacilli Calmette-Guerin regulates leukocyte recruitment by modulating alveolar inflammatory responses

**DOI:** 10.1177/1753425911426591

**Published:** 2012-06

**Authors:** Märta Andersson, Nataliya Lutay, Oscar Hallgren, Gunilla Westergren-Thorsson, Majlis Svensson, Gabriela Godaly

**Affiliations:** 1 Department of Microbiology Immunology and Glycobiology, Division of Laboratory Medicine, Lund University, Lund, Sweden; 2 Department of Respiratory Medicine and Allergology, Division of Clinical Sciences, Lund University, Lund, Sweden; 3 Department of Experimental Medical Science, Division of Vascular- and Respiratory Research Unit of Lung Biology, Lund University, Lund, Sweden

**Keywords:** Leukocyte, mycobacteria, epithelial cells, chemokines, Toll-like receptors

## Abstract

Leukocyte migration into the epithelial compartment is an important feature in the active phase of mycobacterial infections. In this study, we used the Transwell model to investigate the mechanisms behind mycobacteria-induced leukocyte recruitment and investigated the role of TLR2 and TLR4 in this process. Infection of epithelial cells resulted in significantly increased secretion of the neutrophil chemotactic CXCL8 and IL-6, but no secretion of monocyte chemotactic CCL2 or TNF-α was observed. In contrast to epithelial response, mycobacteria-infected neutrophils and monocytes secreted all these cytokines. Corresponding with epithelial cytokine response, mycobacterial infection of the epithelial cells increased neutrophil diapedesis, but decreased monocyte recruitment. However, monocyte recruitment towards mycobacteria infected epithelial cells significantly increased following addition of neutrophil pre-conditioned medium. Mycobacterial infection also increases alveolar epithelial expression of TLR2, but not TLR4, as analyzed by flow cytometry, Western blotting and visualized by confocal microscopy. Blocking of TLR2 inhibited neutrophil recruitment and cytokine secretion, while blocking of TLR4 had a lesser effect. To summarize, we found that primary alveolar epithelial cells produced a selective TLR2-dependent cytokine secretion upon mycobacterial infection. Furthermore, we found that cooperation between cells of the innate immunity is required in mounting proper antimicrobial defence.

## Introduction

Innate host defence mechanisms mounted at an early stage of tuberculosis-infection play a key role in the initiation and direction of adaptive T-cell immunity.^1,2^ Entering the lung, mycobacteria invade the alveolar epithelial cells and resident macrophages that respond by an inflammatory response leading to recruitment of leukocytes from the blood. Leukocyte emigration into the alveolar compartment is an important feature in the active phase of mycobacterial infections. The first phagocyte to arrive to infectious foci is usually the neutrophil that becomes activated by microbial products and chemokines.^3^^–^^6^ Monocytes arrive shortly after and can expand locally into inflammatory macrophages and dendritic cells.^7^ Activation of neutrophils enhances the innate immune defences and leads to the production of antimicrobial peptides, release of granule molecules and extended lifespan of these cells through senescent apoptosis.^8,9^

Monocytes and neutrophils have overlapping expression of pathogen-associated molecular patterns (PAMPs) such as the TLRs.^10^ Ligand binding to TLR initiates a signalling cascade through the recruitment of adaptor molecules leading to a MyD88-dependent and/or a MyD88-independent gene expression.^11^ Epithelial expression of TLR2 and TLR4 during mycobacterial infection is unclear, but mycobacteria activate a variety of cells through these receptors.^6,12,13^ Leukocyte activation through TLR signalling is triggered by mycobacterial products, such as lipoarabinomannan (LAM) and the cell wall-associated and secreted 19-kDa glycolipoprotein.^5,14,15^ Studies in knockout mice revealed that *Tlr2^−^^/^^−^* mice are unable to mount optimal innate and adaptive immune responses against mycobacterial infection, while the effect of *Tlr4* deficiency was less pronounced.^16,17^ However, recent findings suggest that TLR4 plays a protective role in host defence against pulmonary tuberculosis.^17^^–^^19^

Neutrophils are found in abundance in the sputum of tuberculosis (TB) patients and are persistently recruited to sites of chronic mycobacterial infection.^20,21^ CXCL8 is a chemotactic cytokine known for its ability to be a strong neutrophil chemoattractant, but is also chemotactic for monocytes and T-cells.^22,23^ Augmented CXCL8 levels are found in plasma from TB patients and in bronchoalveolar lavage fluids,^24,25^ and this chemokine was shown to enhance neutrophil killing of *Mycobacterium tuberculosis*.^26^ Another chemokine, CCL2, which is a potent chemoattractant and activator for monocytes, also attracts CD4 and γδ T-cells and was shown to play a protective role in murine TB.^27,28^ Pro-inflammatory cytokines, such as IL-6 and TNF-α, orchestrate innate and adaptive host immune responses. IL-6 is required in the rapid expression of an initial protective IFN-γ response during *M. tuberculosis* infection,^29^ while TNF-α is important in the control of mycobacterial infections, as illustrated by the reactivation of TB in the use of anti-TNF drugs.^30^

In the present study, we used a Transwell model system to analyze the recruitment of monocytes and neutrophils to infected alveolar epithelial cells through primary endothelial cells. The recruitment process was quantified by leukocyte diapedesis and by secretion of both pro-inflammatory and inflammatory cytokines. Furthermore, the impact on epithelial TLR2 and TLR4 was investigated in the process.

## Materials and methods

### Bacterial strains and growth conditions

*Mycobacterium bovis* bacilli Calmette-Guerin (BCG) Montreal strain containing the pSMT1 shuttle plasmid was prepared as previously described.^31^ Briefly, the mycobacteria were grown in Middlebrook 7H9 culture medium, supplemented with 10% ADC (Becton Dickinson, Oxford, UK) and hygromycin (50 mg ml^−1^; Roche, Lewes, UK). The culture was dispensed into vials, glycerol was added to a final concentration of 25%, and the vials were frozen at -70°C. Prior to each experiment, a vial was defrosted, added to 9 ml of 7H9/ADC/hygromycin medium and incubated with shaking for 72 h at 37°C. Mycobacteria were then centrifuged for 7 min at 3000 *g*, washed twice with sterile PBS and re-suspended in sterile PBS.

### Cell culture

A549 alveolar epithelial cells (ATCC no. HTB-44) were cultured in Roswell's Park Memorial Institute medium (RPMI) 1640 (Invitrogen, Stockholm, Sweden) supplemented with 0.05 mg ml^−1^ gentamicin (PAA Laboratories, Linz, Austria), 2 mM glutamine and 5% FBS (PAA Laboratories), with a change of medium every third day. The cells were detached by Versene (140 mM NaCl, 2.4 mM KCl, 8 mM Na_2_HPO_4_, 1.6 mM KH_2_PO_4_, 0.5 mM EDTA, pH 7.2).

Primary bronchial epithelial (PBE) cells were used as control to the A549 epithelial cell line throughout the study. Bronchial material for primary cell cultures was obtained from lung explant from healthy donors with no history of lung disease. Bronchial tissue was dissected from lungs and kept in DMEM supplemented with gentamicin, PEST, Fungizone and 10% FBS (all from Gibco, Paisley, UK) until further isolation. After removing intraluminal mucus and surrounding tissue, bronchi were digested in 0.1% protease (Sigma St Louis, MO, USA) prepared in Gibco’s Minimum Essential Medium (S-MEM) supplemented with gentamicin, PEST and Fungizone for 24 h. Bronchial epithelial cells (HBEC) were recovered by repeated intraluminar rinsing with DMEM supplemented with gentamicin, PEST, Fungizone and 10% FBS. Cells were filtered through a 100 µm strainer (Falcon, Becton Dickinson) and seeded in cell culture flasks coated with 1% Collagen−1 (PureCol, Inamed Biomaterial, Freemont, CA, USA) in bronchial epithelial growth medicum (BEGM) cell culture medium (Clonetics, Lonza, Germany). The following day, cells were thoroughly washed with a medium change every other day. Experiments were performed in passage 3. This study was approved by the Swedish Research Ethical Committee in Lund (FEK 91/2006).

Primary HUVECs (Clonetics) were cultured in endothelial cell growth medium (EGM)-2 (Clonetics) supplemented with the EGM-2 bullet kit (hydrocortisone, 0.4% hFGF-B; 0.1% VEGF; 0.1% R3-IGF-1; 0.1% ascorbic acid; 0.1% heparin; 2% FBS; 0.1% hEGFMl and 0.1% GA-1000; Clonetics) (37°C, 5% CO_2_). The cells were used at passages 2–5, according to the manufacturer’s instructions.

Human venous blood mononuclear cells and neutrophils were isolated from healthy volunteers by the Polymorphprep density gradient (Axis-Shield, Oslo, Norway) according to the manufacturer’s instructions as described previously.^32^ Adherent monocytes were obtained by overnight culture on six-well plastic culture plates in RPMI 1640 supplemented with 0.05 mg/ml gentamicin, 2 mM glutamine and 5% FBS at 37°C in a 5% CO_2_ atmosphere. Non-adherent lymphocytes were removed by washing the culture wells with serum-free RPMI 1640 medium.

### Incubation of cells with mycobacteria

For the infection experiments, epithelial A549 or PBE cells were grown in six-well plates (2.0 × 10^5^ cells/well; Fisher Scientific, Loughborough, UK), infected with BCG (one bacterium per cell) or phenol-purified LPS (1 ng ml^−1^; Sigma-Aldrich, Seelze, Germany) at 37°C for up to 5 d. BCG-infected cells and medium alone were used as controls. Neutrophils (2 × 10^6^) or monocytes (1 × 10^5^) were infected with BCG (one bacterium per cell) on a rocking plate at 37°C for 180 min as described previously.^6^ Samples were taken after 0, 60, 120 and 180 min for cytokine measurements.

### Leukocyte migration

HUVEC were grown on Transwells inserts (12-well, 3-µm pore size, Corning, NY, USA) in EGM-2 medium, as previously described.^32^ As a control, cell viability and confluence were analyzed by trypan blue exclusion assay according to manufactures instructions (Sigma-Aldrich). Transwell model system was created by combining primary endothelial cells in the upper inserts and infected alveolar A549 or PBE epithelial cells in the lower compartment. For the blocking experiments, monoclonal mouse anti-human TLR2 and/or monoclonal mouse anti-human TLR4 Abs (R&D Systems, Copenhagen, Denmark) 10 µg/ml were added to the Transwell system or to the individual cell groups 30 min before the addition of BCG.

Leukocyte transmigration assays were performed as previously described.^32^ Briefly, neutrophils (1.5 × 10^6^, 0.5 ml) or monocytes (1.0 × 10^5^, 0.5 ml) were added onto the endothelial cells in upper well. For the experiments on neutrophil and monocyte interactions, monocytes were added to the upper well 3 h after the addition of neutrophils [past neutrophils (PN)] or in the presence of medium from BCG infected neutrophils [pre-conditioned medium (PCM)] in the lower compartment. After 3 h, samples were taken from the bottom well for leukocyte counting by Cell Counter (Sysmex, Hamburg, Germany) and analyzed for cytokines.

### Flow cytometry

Epithelial TLR2 and TLR4 expression was investigated by FACS analysis. Alveolar epithelial A549 cells were infected as mentioned above and stained with monoclonal mouse anti-human TLR2 or monoclonal mouse anti-human TLR4 Abs (10 µg ml^−1^; R&D Systems) for 30 min on ice. The PBE cells were used as a control. The epithelial cells were washed twice in PBS and stained with secondary anti-mouse IgG-FITC Abs (10 µg ml^−1^; Autogen Bioclear UK, Calne, UK) for 30 min on ice. FITC-labelled monoclonal mouse immunoglobulin G (10 µg ml^−1^; mouse IgG isotype control, R&D Systems) was used as a negative control. Cells were washed twice in PBS and analyzed by flow cytometry in a FACS Calibur instrument (Becton Dickinson). A total of 5000 cells were counted in each sample. Mean fluorescence intensities were calculated from the uninfected control.

### Confocal microscopy

Epithelial expression of TLR2 and TLR4 was detected by confocal microscopy. After infection, the alveolar epithelial A549 cells were spun down on glass slides in a cytospin2 centrifuge (RP centrifuge; HettichRotana, Malmö, Sweden) at 600 rpm for 5 min and fixed in 100% methanol. Before staining, the cells were permeabilized with 0.25% Triton-X (VWR International, Stockholm, Sweden) in PBS with 5% FBS for 10 min. Slides were incubated with anti-TLR2, anti-TLR4 or control IgG Ab (10 µg/ml; mouse IgG isotype control, R&D Systems) for 40 min at room temperature (23°C). The slides were washed in PBS and incubated with secondary goat anti-mouse IgG Abs (Alexa Flour 488, Invitrogen, Carlsbad, CA, USA) in a dilution of 1:200 for 40 min in the dark at room temperature. Staining of DNA was performed by incubating the cells with propidium iodide (1 mg/ml) for 15 min in the dark at room temperature. After washing in PBS, the slides were examined with LSM-510 confocal equipment (Carl Zeiss, Oberkochen, Germany).

### ELISA

CXCL8, CCL2, IL-6 and TNF-α secretion by the infected cells was quantified in supernatants by ELISA (R&D Systems) according to the manufacturer’s instructions.

### Western blot

The epithelial cells were washed with PBS containing 0.2 mM phenylmethylsulphonyl fluoride (PMSF), 1 lg ml^−1^ PepstatinA, 5 lg ml^−1^ Leupeptin (Sigma-Aldrich) and complete protease inhibitor cocktail (Roche Diagnostics, Mannheim, Germany), and lysed in modified radio-immunoprecipitation assay (RIPA) buffer (50 mM HEPES, 150 mM NaCl, 2 mM EDTA, 50 mM ZnCl, 1% NP-40, 0.1% deoxycholate, 0.1% SDS) containing the same protease inhibitors. Protein concentrations were measured with the DC Protein Assay (Bio-Rad Laboratories, Hercules, CA, USA). Equal amounts of protein were separated by SDS-PAGE and blotted onto polyvinylidene fluoride (PVDF) membranes. Membranes were saturated with non-fat, dry milk and incubated with mouse mAb against TLR2 (R&D Systems) or TLR4 (R&D Systems) (all 1:500–1000) or mouse anti-GAPDH Ab (1:3000–5000; Novus Biologicals, Littleton, CO, USA). Bound Abs were detected with rabbit anti-mouse HRP-conjugated Ab (1:50,000–200,000, Novus Biologicals) using ECL plus Western blotting reagent (GE Healthcare, Little Chalfont, UK) and GelDoc equipment (Bio-Rad Laboratories). To quantify protein levels, band intensity was measured with ImageJ software28 and normalized against GAPDH. If required, membranes were stripped with Restore Western Blot Stripping Buffer (Pierce, Rockford, IL, USA), blocked and re-probed with new Abs.

### Statistical analysis

The statistical difference was investigated by Mann-Whitney test (***P ≤ 0.001, **P < 0.01, *P < 0.05, ns = non significant).

## Results

### Selective leukocyte recruitment

**Figure 1.**
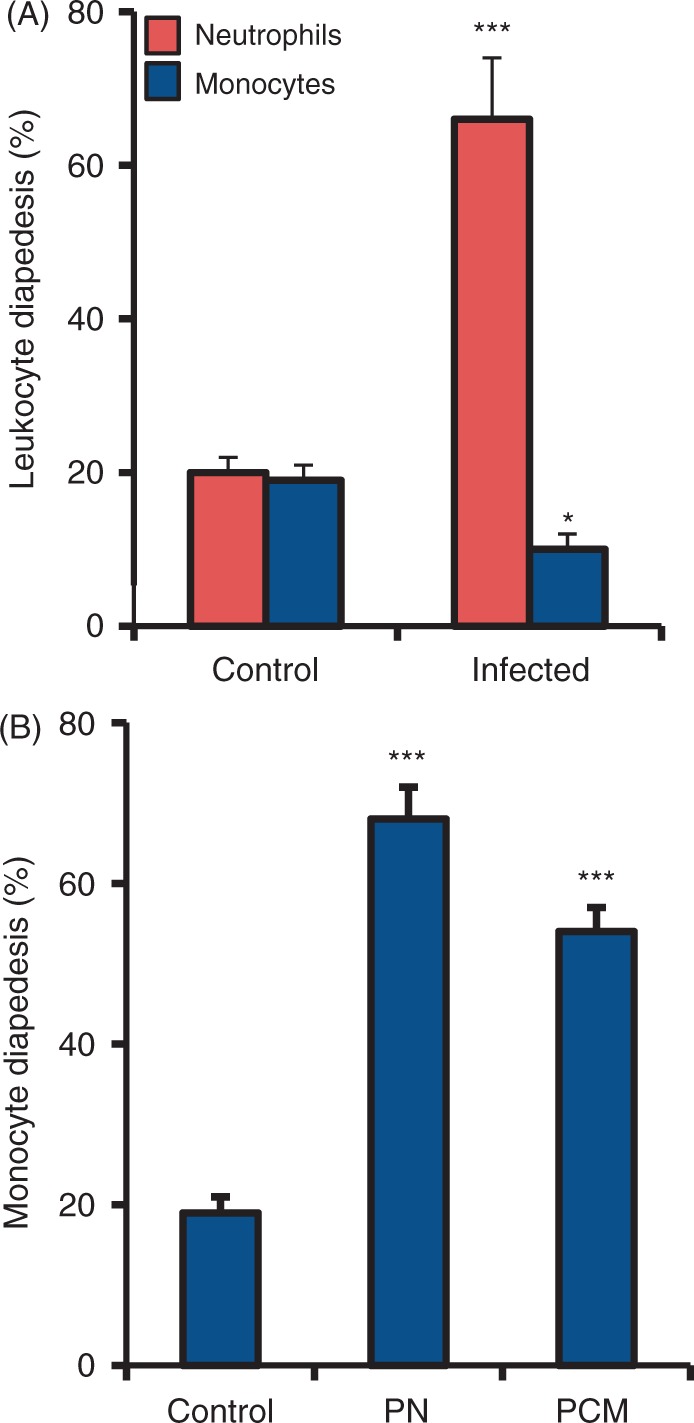
Neutrophil diapedesis increases monocyte recruitment. Mechanisms of leukocyte recruitment during mycobacteria-induced infection in the Transwell model. (A) Normal leukocyte diapedesis after 3 h across unstimulated endothelial cells towards unstimulated alveolar epithelial cells was below 20% of the total amount added. For the infection model, alveolar epithelial cells were infected with BCG for 3 d whereupon they were combined with the Transwell inserts containing primary endothelial cells, and neutrophils or monocytes were added. BCG infection of the epithelial cells significantly increased neutrophil recruitment across the endothelial cell layer while monocyte migration was decreased. (B) Addition of monocytes after the passage of neutrophils (PN) or after the addition of pre-conditioned medium (PCM) from neutrophils in the bottom well increased monocyte recruitment. Data are presented as mean ± SEM of four separate experiments; *P < 0.05, *** *P* < 0.001, compared to non-infected control.

Mycobacterial invasion of alveolar epithelial A549 and PBE cells leads to secretion of chemokines CXCL8 and CCL2, but the initial events of leukocyte recruitment are not known.^33^ The Transwell model was used to investigate the mechanisms of leukocyte recruitment during mycobacteria-induced infection. Normal leukocyte diapedesis across unstimulated endothelial cells towards unstimulated epithelial cells was below 20% of the total amount added (Figure 1A). However, BCG infection of the epithelial cells significantly increased neutrophil recruitment across endothelial cell layer from 20% to 66% (*P* ≤ 0.001), while monocyte migration was decreased to 10% (*P* = 0.016). To investigate if neutrophil recruitment could affect monocyte migration, monocytes were added 3 h after the addition of neutrophils to endothelial barrier during mycobacterial epithelial infection. Monocyte recruitment after neutrophil diapedesis increased from 20% to 68% (*P* ≤ 0.001; Figure 1B). To analyze whether the increased monocyte migration was dependent on the disruption of endothelial integrity, monocyte diapedesis was measured in response to the addition of medium from neutrophil experiments and BCG infected epithelial cells (Figure 1B). Normal neutrophil diapedesis across unstimulated endothelial cells was 18% of the total amount added (Figure 1B). Addition of pre-conditioned medium increased monocyte migration from 18% to 54% (*P* ≤ 0.001).

### Selective cytokine secretion

**Figure 2.**
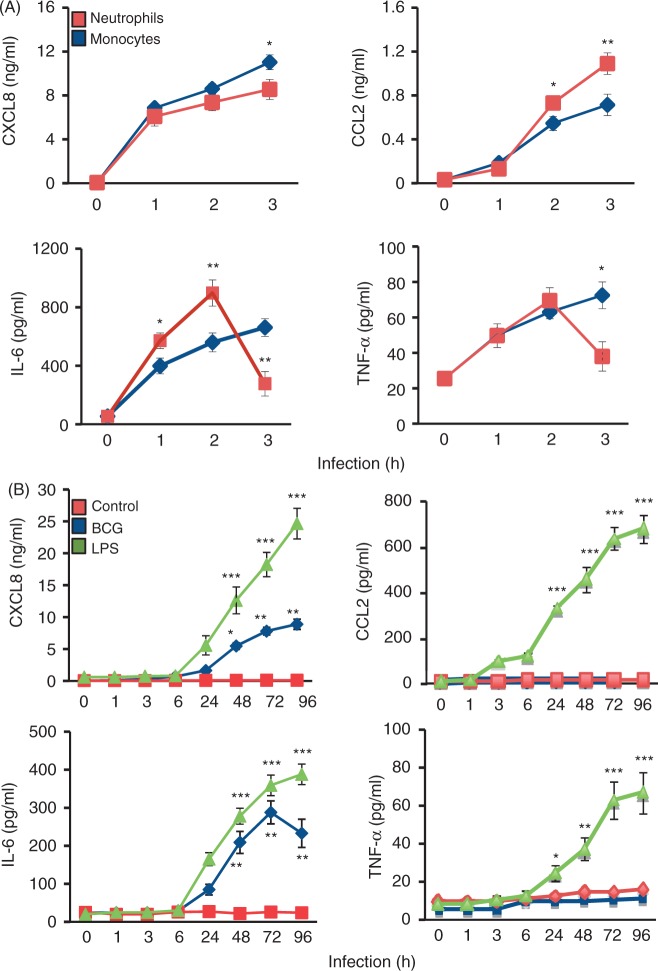
Mycobacteria induce a selective cytokine secretion. Induction of cytokine secretion by BCG-infected human leukocytes was measured for up to 3 h, while cytokine secretion from mycobacterial infected alveolar epithelial cells was measured over 96 h. (A) Compared with non-infected control cells, mycobacterial infection of neutrophils and monocytes induced significant CXCL8, CCL2, IL-6 and TNF-α secretion. After 2 h of infection, neutrophil CCL2 and IL-6 secretion were significantly higher than monocyte secretion of these cytokines; however, mycobacterial infection of monocytes induced a continuous and persistent pro-inflammatory cytokine production. (B) Alveolar epithelial response of CXCL8 and IL-6 after mycobacterial infection was delayed. CXCL8 secretion peaked after 72 h while there was a continuous increase in IL-6 secretion during the study. Mycobacteria did not induce CCL2 or TNF-α production from infected alveolar epithelial cells. LPS was used as a positive control and non-infected cells were used as negative control. Data are presented as mean ± SEM of four separate experiments; *P < 0.05, **P < 0.01, ***P < 0.001, neutrophils compared to monocytes in (A) and compared to non-infected control in (B).

Effective cytokine secretion is essential in order to construct an appropriate inflammatory response against an infection. Induction of inflammatory response by mycobacteria-infected neutrophils, monocytes and epithelial A549 cells was estimated as a concentration of chemokines CXCL8 and CCL2 and of the pro-inflammatory cytokines IL-6 and TNF-α (Figure 2). The PBE cells were used to compare mycobacteria-induced cytokine production. Compared with medium control, mycobacterial infection of neutrophils and monocytes induced an ascendant CXCL8 and CCL2 secretion that peaked 3 h after infection (Figure 2A). Neutrophil CCL2 secretion was significantly higher than monocyte secretion 2 h after infection (*P* = 0.029 after 2 h; *P* < 0.001 after 3 h). There was a difference between neutrophils and monocytes in the pro-inflammatory cytokine response to mycobacterial infection. Neutrophil infection induced an IL-6 and TNF-α production that peaked 2 h after infection and declined thereafter. However, mycobacterial infection of monocytes induced a continuous pro-inflammatory cytokine production during the whole experiment. The secretion of CXCL8 and IL-6 from infected epithelial cells was delayed and began 24 h of infection (Figure 2B). No difference in cytokine production or cytokine secretion was observed between the alveolar A549 and the PBE epithelial cells. CXCL8 secretion peaked after 72 h, while there was a continuous increase in IL-6 secretion during our study. Mycobacteria did not induce CCL2 or TNF-α production from infected primary epithelial cells, while LPS, used as a positive control, increased all cytokines (*P* < 0.001)

### Mycobacteria increase TLR expression

**Figure 3.**
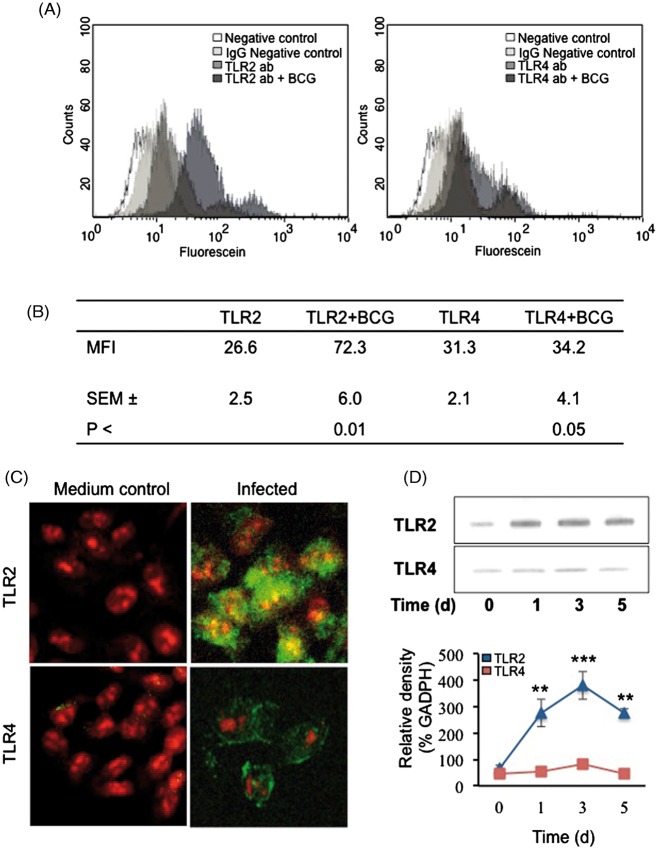
Mycobacteria increase alveolar TLR expression. TLR2 and TLR4 expression on alveolar epithelial cells. (A) Alveolar epithelial cells were infected with mycobacteria for 3 d and stained with monoclonal anti-TLR2 or anti-TLR4 Ab and expression was analyzed with flow cytometry (A,B) and confocal microscopy (C). Unstimulated alveolar epithelial cells have low basal level expression of both receptors, with TLR4 more abundant than the TLR2. The mean fluorescence intensity (MFI) was calculated as an increase from negative control cells. Mycobacterial infection significantly increased epithelial TLR2 expression as detected by confocal microscopy and quantified by flow cytometry, but TLR4 expression was less affected. Confocal images showing alveolar epithelial TLR2 or TLR4 expression after 3 d of mycobacterial infection (green). Cellular DNA was counter-stained with propidium iodide (red). Original magnification x 300. (D) Alveolar epithelial TLR2 and TLR4 expression upon mycobacterial infection for up to 5 d was analyzed by Western blot. Epithelial cells express continuous TLR2 that was further increased in response to mycobacterial infection, while TLR4 expression was not affected. Data are presented as mean ± SEM of four separate experiments; **P < 0.01, ***P < 0.001, TLR2 expression compared to TLR4 expression.

TLR2 and TLR4 are expressed on various cells in the lung,^34^ but their expression on human lung alveolar epithelial cells has not been investigated. To address this question, alveolar A549 cells were stained with monoclonal anti-TLR2 or anti-TLR4 Abs (Figure 3A, B) and the surface protein expression was quantified by flow cytometry. The PBE cells were used to compare mycobacteria-induced TLR2 and TLR4 expression. Unstimulated A549 and PBE epithelial cells had low basal level expression of both receptors, with the TLR4 more abundant than the TLR2. Mycobacterial infection significantly increased epithelial TLR2 expression, as detected by confocal immunofluorescent microscopy and quantified by flow cytometry; however, TLR4 expression was less affected (Figure 3A–C). Mycobacterial infection augmented TLR2 expression by 52% and the TLR4 expression was increased by 17%. These results were further confirmed by Western blot analysis that revealed continuous increased expression of TLR2 in both A549 and PBE cells in response to mycobacterial infection; however, TLR4 expression was not affected (Figure 3D). Infected cells had an increased expression level of TLR2 for up to 5 d after stimulation (*P* = 0.008 for TLR2 expression).

### TLRs and neutrophil diapedesis

**Figure 4.**
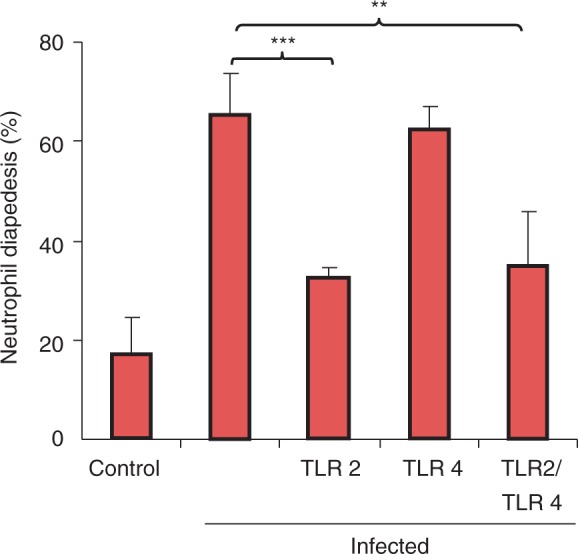
TLR2 is important for neutrophil diapedesis. The impact of TLR2 and TLR4 on neutrophil diapedesis was investigated with the Transwell model. Alveolar epithelial cells were infected for 3 d with mycobacteria. After infection, epithelial cells were combined with endothelial cells and human neutrophils in the Transwell model and neutrophil diapedesis was recorded in the lower compartment after 3 h. Blocking with anti-TLR2 Abs or with both TLR2 and TLR4 Abs induced a significant decrease of mycobacteria-induced neutrophil diapedesis. Blocking of TLR4 did not have any impact. Data are presented as mean ± SEM of four separate experiments; **P < 0.01, ***P < 0.001, compared with infected control.

Mycobacteria are known to trigger leukocyte activation by engaging TLR2 and TLR4,^5,14^ but not much is known about the impact of these receptors on leukocyte diapedesis. The Transwell model was used to investigate the influence of TLRs on neutrophil migration across activated endothelium towards infected A549 or PBE epithelium (Figure 4). Blocking with anti-TLR2 Abs induced a significant decrease of mycobacteria-induced neutrophil diapedesis (from 66% to 32%; *P* < 0.001), while the blocking of TLR4 did not have any impact. Blocking of both receptors reduced neutrophil diapedesis from 66% to 35% (*P* = 0.019).

### TLR2 modulated cytokine secretion

**Figure 5.**
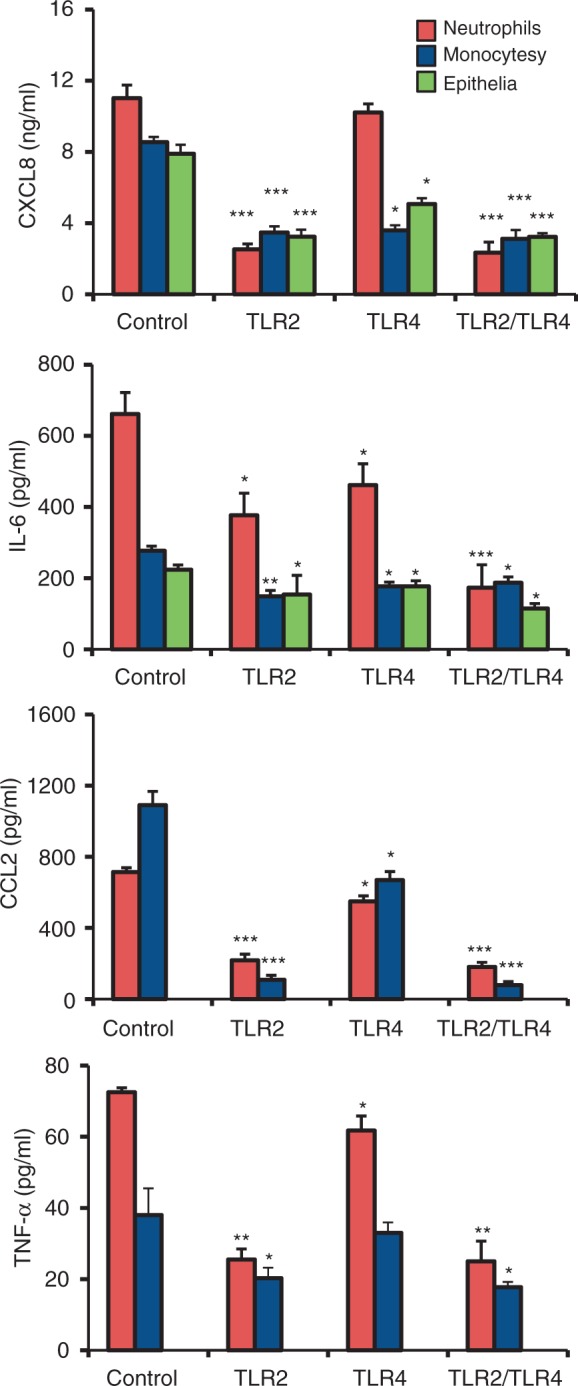
TLR2 control cytokine secretion. Influence of TLR2 and TLR4 on mycobacteria induced cytokine secretion from neutrophils, monocytes and alveolar epithelial cells. The cells were treated with Abs against TLR2 and/or TLR4 before infection with live BCG. Secretion of CXCL8, IL-6, CCL2 and TNF-α was measured after 3 h for leukocytes and after 3 d for epithelial cells. Blocking of TLR2 significantly reduced the cytokine secretion of all the infected cells, while blocking of TLR4 had the highest inhibitory function on epithelial and monocyte cytokine secretion. Blocking of both receptors significantly reduced cytokine secretion of all the infected cells. Data are presented as mean ± SEM of four separate experiments; *P < 0.05, **P < 0.01, ***P < 0.001, compared with infected control.

TLRs modulate the induction of hundreds of host genes through a complex network of signalling that allows for the appropriate response to a microbial pathogen.^35^ In order to investigate how TLR2 and TLR4 influence mycobacteria induced cytokine secretion from neutrophils, monocytes, alveolar A549 and PBE epithelial cells, the cells were treated with Abs against TLR2 and/or TLR4 before infection with live BCG (Figure 5). Blocking of TLR2 significantly reduced the cytokine secretion of all the infected cells, while blocking of TLR4 had the highest inhibitory function on both epithelial cells tested and on monocyte cytokine secretion. Blocking of both receptors significantly reduced cytokine secretion of all infected cells.

## Discussion

Mycobacteria have evolved a highly specific mechanism to proliferate in the host during infection. By manipulating the important host defence mechanisms, such as apoptosis and phagocytosis, this pathogen has killed an estimated 100 million people over the last century.^36^ In this study, we show that mycobacteria induce a specific inflammatory response from infected epithelium resulting in selective leukocyte recruitment. Mycobacterial infection of alveolar epithelial cells resulted in secretion of CXCL8 and IL-6, but no secretion of the monocyte chemotactic CCL2 or pro-inflammatory TNF-α was observed. Both of the epithelial cells used in our study, i.e. alveolar and bronchial, exhibited an identical inflammatory response against mycobacterial infection. However, in a human lung, the epithelial inflammatory response against mycobacteria would also be affected by the normal macrophage:epithelia ratio of 50:1000,^37^ which is missing in our Transwell model system. The epithelial response of CXCL8 would lead to recruitment of mainly neutrophils from the blood circulation, which was further confirmed in the model system. Infection of epithelial cells increased neutrophil diapedesis, while there was a decrease of monocyte recruitment. However, the addition of pre-conditioned medium from infected neutrophils increased monocyte recruitment, suggesting that arriving neutrophils secrete chemokines to attract more cells to the site of infection.

Analysis of the cytokine repertoire from mycobacteria-infected neutrophils and monocytes revealed that both of these cells secrete the measured cytokines, i.e. CCL2, CXCL8, TNF-α and IL-6. Interestingly, administration of the cytokines IL-6 and TNF-α was recently shown to induce a protective T cell immunity against *M. tuberculosis* infection.^38^ Monocyte secretion of CCL2, CXCL8, TNF-α and IL-6 was more persistent and increased over time, while neutrophil cytokine response declined by the end of our study, possibly reflecting the short lifespan of these cells. Neutrophils have been implicated in anti-mycobacterial immunity based on the transfer of antimicrobial molecules to monocytes.^39^^–^^41^ Both apoptotic neutrophils and purified neutrophil granules can reduce the viability of bacteria and augment the ability of infected macrophages to reduce bacterial growth. Neutrophil protection against infection was recently assessed in newly diagnosed TB patients.^42^ In this study, a depletion of neutrophils from a whole blood model dramatically reduced the release of anti-mycobacterial peptides in response to mycobacterial infection and reduced the ability to limit mycobacterial viability. Importantly, an inverse relationship was found between the amount of peripheral neutrophils and the risk of *M. tuberculosis* infection, suggesting that neutrophils play a protective role very early on in infection.^42^ Additionally, another leukocyte, the eosinophil, is found in high amounts in serum of TB patients, but their function in immune response towards mycobacterial infections is not well investigated.^43^

In the lung, TLR2 and TLR4 are mainly expressed on the surface of monocytes and neutrophils,^34^ but the mRNA level of these receptors within alveolar epithelial cells has been reported.^44^^–^^46^ We observed that mycobacteria induced increased TLR2 expression, while TLR4 expression was less affected by mycobacterial infection. This observation was further supported by protein analysis, where we could observe that epithelial TLR2 levels increased upon infection, while TLR4 levels remained at the background levels. Blocking of TLR2 had a major impact on neutrophil migration across activated endothelia, while blocking of TLR4 had no significant effect. One explanation could be that TLR signalling regulates neutrophil migration by increasing cell surface expression of chemokine receptors and activating CD11b/CD18.^47,48^ Chemokine secretion is also affected by TLRs, as blocking of TLR2 effectively decreased mycobacteria-induced cytokine secretion from both leukocytes and alveolar epithelial cells, while the blocking of TLR4 had the highest inhibitory function on epithelial and monocyte cytokine secretion. This observation is supported by previous studies on mycobacterial cell activation where secretion of CXCL8 was dependent on cooperation between TLR2 and TLR4.^6,13^ Furthermore, the importance of intact TLR2 signalling was demonstrated by the increased susceptibility to TB infection in patients with *TLR2* polymorphisms.^49,50^

In summary, we found that primary alveolar epithelial cells produced a selective, TLR2-dependent cytokine secretion upon mycobacterial infection. Most importantly, we found that neutrophil influx is necessary for increasing the inflammation and recruitment of monocytes by broadening the repertoire of the inflammatory response. Cooperation between cells in innate immunity is thus required in mounting a proper antimicrobial defence.
